# Similarities and differences of polyadenylation signals in human and fly

**DOI:** 10.1186/1471-2164-7-176

**Published:** 2006-07-12

**Authors:** Dorota Retelska, Christian Iseli, Philipp Bucher, C Victor Jongeneel, Felix Naef

**Affiliations:** 1 Swiss Institute of Bioinformatics, Batiment Genopode, UNIL, 1015 Lausanne, Switzerland; 2 Swiss Institute for Experimental Cancer Research (ISREC), Ecole Polytechnique Fédérale de Lausanne (EPFL), AAB-021, CH-1015 Lausanne, Switzerland; 3 Ludwig Institute for Cancer Research, Batiment Genopode, UNIL, 1015 Lausanne, Switzerland

## Abstract

**Background:**

Cleavage of messenger RNA (mRNA) precursors is an essential step in mRNA maturation. The signal recognized by the cleavage enzyme complex has been characterized as an A rich region upstream of the cleavage site containing a motif with consensus AAUAAA, followed by a U or UG rich region downstream of the cleavage site.

**Results:**

We studied these signals using exhaustive databases of cleavage sites obtained from aligning raw expressed sequence tags (EST) sequences to genomic sequences in *Homo sapiens *and *Drosophila melanogaster*. These data show that the polyadenylation signal is highly conserved in human and fly. In addition, *de novo *motif searches generated a refined description of the U-rich downstream sequence (DSE) element, which shows more divergence between the two species. These refined motifs are applied, within a Hidden Markov Model (HMM) framework, to predict mRNA cleavage sites.

**Conclusion:**

We demonstrate that the DSE is a specific motif in both human and *Drosophila*. These findings shed light on the sequence correlates of a highly conserved biological process, and improve *in silico *prediction of 3' mRNA cleavage and polyadenylation sites.

## Background

The process of mRNA 3' end formation requires pre-mRNA cleavage followed by polyadenylation. Recent reports support the hypothesis that cleavage occurs during transcription and directly influences transcription termination in yeast and human cells [1,2]. mRNA cleavage in mammals is thought to be controlled by two dominant sequence signals, the well defined polyadenylation signal (PAS) located 10–30 bases upstream of mRNA cleavage site (CS) and a less conserved U-rich sequence, called downstream sequence element (DSE) and located within the first 30 nucleotides downstream of the CS. Protein complexes involved in cleavage and polyadenylation have been identified: the PAS is bound by the cleavage and polyadenylation specificity factor (CSPF), while the DSE recruits the cleavage stimulation factor (CstF) (reviewed in [3]).

In mammals, the PAS has been identified early on from its high degree of conservation [4] and studies have shown that point mutations of the consensus sequence AAUAAA decrease or abolish polyadenylation efficiency [5]. However, genome-wide analysis of PAS clearly shows that some variants which are less efficient *in vitro *are functional *in vivo *[6,7]. The second important region, the DSE, is less conserved in mammalian genes and no clear consensus signal has been defined [3]. Tabaska and Zhang suggested a consensus motif based on motif searches in ~100 test sequences [8]. Counting overrepresented words identified U-rich hexamers in genome-aligned fly ESTs [25], but failed to identify a conserved DSE motif in human[9]. Selex experiments reported binding of CstF to a small number of related sequences (reviewed in [3]), while NMR studies suggested that a stretch of two adjacent uridines is crucial for CstF binding [10].

The position of the CS was shown to be primarily defined by the distance between the PAS and the DSE, and to occur preferentially 5' of an adenine [11]. Survey of Unigene clusters show that the positions in the CSs of individual ESTs in a cluster tend to vary over a distance of 30–40 nucleotides [12]. This micro-variability in the cleavage position further suggests that the fixed anchors governing the cleavage process are the PAS and DSE and not the CS itself.

Accurate characterization of 3' termination is particularly relevant for gene prediction programs. To define the 3'-ends of genes, the HMMgene program scans for the occurrence of the AAUAAA hexanucleotide (A. Krogh, personal communication) while Genscan [13] applies a simplified model of the mRNA cleavage site that uses an approximate scoring for the PAS irrespective of the presence of a DSE. Thus, these programs are prone to false positives and ignore mRNA CSs that do not match this motif. Attempts to take into account other sequence features of the CS region in human sequences used different computational approaches. In a program called poladq, Tabaska and Zhang [8] included the DSE motif using a quadratic discriminant function. Legendre and Gautheret [9] designed a weight matrix based on the nucleotide composition in the 46 bases following the PAS. Both programs search the genomic sequence for the occurrence of a PAS, and then scan the putative positives for further signals. Recent reports have successfully demonstrated the applicability of Hidden Markov Models (HMMs) to the problem in *S. cerevisiae *[14] or *C. elegans *[15].

Here, we take advantage of the systematic mapping of the raw data from >1'500'000 human 3'ESTs [16] and of >30'000 *Drosophila *3'ESTs to their respective genomes to model and compare PAS and DSE signals in both species using a HMM framework. This study establishes that the main features of mRNA cleavage regions are clearly conserved between both species, with a highly conserved PAS signal and the presence of a DSE in both species. Model assessment showed that including the DSE in HMMs significantly improves the accuracy of CS predictions.

## Results

### 3'UTR length

**Figure 1:**
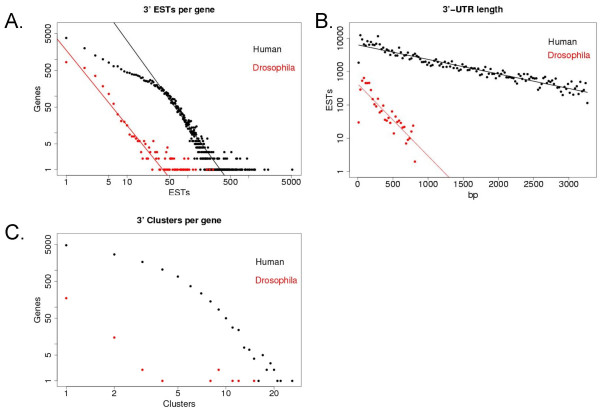
Distribution of 3' EST tags in human and *Drosophila melanogaster *genomes. A: Distribution of trusted tags per gene. In both species, the distribution of tags per gene follows a power law, with similar exponents (-2.29 for human tags and -2.05 for *Drosophila *tags). B: 3'UTR length distributions, in human and Drosophila genes, the average length for humans is 995 bp, for *Drosophila *it is 200 bp. Each point is the number of 3'UTRs of given length. C: Number of polyadenylation clusters in genes having more than 1 cluster, human genes have up to 25 separate 3P clusters (mean number of clusters per human gene is 2, 3, for drosophila genes 1.11).

We compared the length of human and *Drosophila *3'-UTRs defined as the distance between the stop codon and the CS (Figure 1B). To avoid ambiguities, only sites from genes with a unique stop codon, documented by at least one entry in RefSeq were included. Both length distributions are good exponentials with characteristic lengths of 995 bases in human and 200 bases in *Drosophila*.

### Polyadenylation signal variants

We investigated the frequencies of PAS variants reported in [6], in the 50 bp upstream of CS documented by 3' tags. Tags with matches to multiple variants were attributed to the most common variant, so that each tag counted only once. 46.7% of human and 47.7% of *Drosophila *sequences had the main PAS variant AAUAAA, while 16% (10%) had the second motif AUUAAA. Less common motifs were present within both species, but interestingly 15% of human *bona fide *cleavage sites (22.47% in *Drosophila*) had none of the previously reported PAS motifs (Table 1). This suggests that cleavage can be induced by yet unknown signals and/or mechanisms. However, we were unable to find such signals in this dataset.

### Compositional bias near mRNA CSs

**Figure 2:**
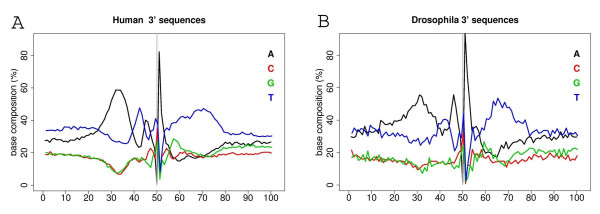
Nucleotide composition profile is conserved between human and Drosophila cleavage sites. A: Human ESTs (590008). B: *Drosophila *ESTs (11385). CS at position 50 is marked by a gray line.

To examine the nucleotide composition of all polyadenylation regions, we considered for each site the 100 bases consisting of the 50 bases upstream of the CS followed by 50 bases from the genomic sequence. Collected tags are then aligned on the position of mRNA cleavage occurring between position 50 and 51, following reports showing that mRNA cleavage occurs preferentially 5' of an A [11]. If the nucleotides immediately 5' of the mRNA CS are one or several A's, the mapping procedure causes an unavoidable ambiguity in the real position of the cleavage. This is reflected by the absence of A's at position 50 and the amplification of the sharp peak in position 51. Nevertheless, simulations show that the human cleavage regions (Figure 2A) have significantly higher A frequency at position 51 (~80%) than random sequence with identical nucleotide composition (not shown).

Other noticeable features include an A-rich region 15–25 bases upstream of the CS (positions 20–35 in Figure 2A, encompassing the PAS), and a U-rich region 10–25 bases downstream of the CS (positions 60–75). The A-rich region upstream of the CS is followed by a narrower (~5 nt) U-rich peak, then a small A-rich region, and possibly another U-rich peak immediately before the mRNA CS. An increase in Gs is visible between 5 and 10 nucleotides after the cleavage site, partly overlapping with the broad U-rich peak.

Interestingly, all features described above are conserved in *Drosophila *(Figure 2B). The A frequency at position 51 reaches 90%, which is noticeably higher than in human regions. We computed the average A frequency in the 10 bases encompassing mRNA CS. In human the A frequency in this region is 32,3%, which is close to the average sequence composition of the polyadenylation regions. *Drosophila *sequences show a local increase to 45,4% A's near the CS compared to a 34,25% average A content. The coarse compositional bias in the mRNA cleavage region is clearly conserved between both species, pointing to a functional conservation of mRNA cleavage and possibly a participation of several observed regions in the cleavage process.

### Sequence motifs near cleavage sites

**Figure 3:**
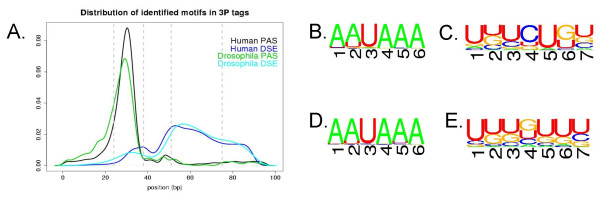
Position and consensus sequence for PAS and DSE identified in Human and *Drosophila *cleavage site sequence tags (A). Dotted lines: Windows defining the subset of motifs used for the final weight matrix determination, pos 24–38 for PAS and 51–75 for DSE (CS is at position 50). B-E: sequence logos for the inferred weight matrices. B: Human PAS. C: Human DSE. D: *Drosophila *polyadenylation signal. E: *Drosophila *DSE.

Using unsupervised motif searches, we investigated whether specific sequence motifs would further characterize the neighborhood of the CS. For humans (respectively *Drosophila*) 3'UTR tags were searched in groups of 250 (cf. Methods) and two dominant signals were identified. Figure 3A shows the positional bias of both signals and this information was incorporated in our definitions of the weight matrices (cf. Material and Methods and Figure 3).

AAUAAA was unambiguously found as the dominant signal in both species, followed by weaker U-rich signals downstream of the CS. No other motifs occurred systematically. The human polyadenylation signal (3B) shows a very strong AAUAAA consensus, with a substitution of A to U at position 2 occurring in ~20% of the cases, consistent with observed frequency of the AUUAAA variants (table 1). The *Drosophila *PAS (Figure 3D) is remarkably similar (details in Table 2), and the differences reflect the slight variations in proportion of different variants shown in table 1. Although the significance of the second motif (DSE) was marginal, its consistency across independent sets and positional bias suggested that we identified a putative human DSE (Figure 3C). Notice that this DSE seems related to that identified by Tabaska & Zhang [8], albeit shorter by 3 nucleotides. Information content (cf. Methods) did not support this extension in our dataset. Scanning by information content (cf. Methods) indicated that a longer descriptor did not improve performance in our dataset. The second motif identified in *Drosophila *is also U-rich, and similarly positioned relative to mRNA CS as the human DSE (Figure 3A). However, the profile is clearly different (Figure 3E). Based on its sequence and nucleotide composition, we refer to this motif as the *Drosophila *DSE.

### HMM model for polyadenylation sites

**Figure 4:**
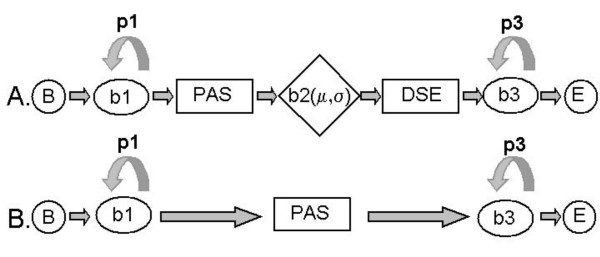
HMM models used to predict mRNA termination. A: Double model used in specificity tests and mRNA CS predictions (Figure 5). Both PAS and DSE separated by a Gaussian spacer must be used. Zero^th^-order emission probabilities are b1 and b3, and self-transitions p1 and p3. In the Gaussian spacer the emission probability is b2. In both models b1 = b2 = b3. B: PAS-only model: control model including a single weight matrix.

To establish whether the DSE element would help to predict cleavage sites, we constructed a HMM model for the detection of polyadenylation sites in genomic sequences (Figure 4). The model uses the two matrices, linked by a spacer with Gaussian length distribution of mean 40 bases and standard deviation (SD) 11 in human and *Drosophila*. These parameters are close to those estimated from Figure 3 and showed optimal behavior in the predictions. We investigated several other sequence features, such as dinucleotide frequencies at the CS, extended PAS and DSE matrices, or different background emission probabilities in each background region, including first order dinucleotide background models. With the exception of the second matrix, we found no evidence that any of these additions would improve polyadenylation site prediction using our evaluation procedures (see below). We therefore restricted further study to the one (PAS-only, Figure 4B) versus two matrix (Double) model (Figure 4A).

Model parameters were optimized and performances were evaluated using two different approaches, one in which we compared optimal scores (Forward decoding) for real polyadenylation sites-containing sequences with scores obtained from randomized sequences, and the other in which we evaluated the performance of the model in predicting the positions of documented polyadenylation sites.

### Specificity assessment by comparison to randomized sequences

**Figure 5:**
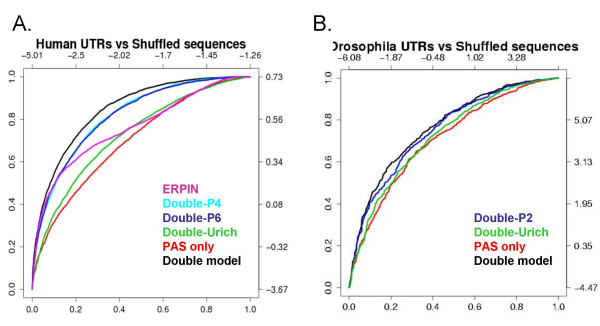
The double model including the deduced DSE matrices improves the discrimination of real 3'UTRs from control sequences. ROC curves showing comparison of best scores (Forward decoding). True positive rate was computed for 3'880 3'000 bp long human sequences (A) and 840 600 bp long Drosophila sequences (B). False positive rate was computed on the test set sequences randomized in 100 bases windows. A-B: Double model (black curve) is more specific than PAS-only model (red), and that ERPIN 3.1 predictions. The DSE matrix included in the double model allows better recognition than a U-rich 25 nt matrix (green) or modified versions of the DSE, where position 4 or 6 (human) or 2 (Drosophila) have been replaced with background nucleotide frequency.

Our aim was to confirm that the identified DSE elements improved prediction of mRNA cleavage sites. To this end, the HMM models shown in Figure 4A and 4B were optimized on training sequences consisting of 3000 bases downstream of unique stop codons in humans (600 for *Drosophila*; cf. Methods). Receiver Operating Characteristics (ROC) analysis was used to compare predictions from the various models (Figures 5A and 5B, black curves). In both human and *Drosophila*, the models including both PAS and DSE weight matrices were clearly more specific than the single weight matrix model. The ERPIN program [9] achieved optimal predictions using the 46 bp downstream of the PAS, which do not systematically include the DSE according to our distance estimates. The comparison of our model to ERPIN 3.1, using our test sets and following the protocol described by the authors [9] confirms the higher specificity of our model.

Furthermore, to assess the specificity of the DSE weight matrices, we substituted the DSE matrix with U-rich profiles. The DSE matrix was replaced with a profile of the whole U-rich region (bases 55 to 80, see figure 2A and 2B). The use of this 25-nt weight matrix decreases the specificity of polyadenylation site prediction in both human and Drosophila 3'UTRs (Figure 5A and 5B, green curve). Both DSE profiles include specific positions that have strong preferences for non-U (C or G) nucleotides (figure 3C and 3E). To assess whether these significantly contributed to the DSE specificity, positions 4 and 6 in the human DSE matrix and position 2 in *Drosophila *DSE were replaced with background nucleotide frequency. Each of these small changes of the DSE profiles resulted in a systematic decrease in specificity (Figure 5A and 5B, blue curves).

### Prediction of 3' mRNA termination

Since mRNA cleavage is thought to occur at the 5'-most site having a sufficiently strong cleavage signal [3], we designed a prediction paradigm that mimics the biological cleavage process. After model optimization (cf. below), we computed the posterior label probability (PLP) of the PAS for each nucleotide in each human test sequence. To measure the sensitivity and specificity of the predictions, a range of thresholds in PLP was chosen. For each fixed threshold and each sequence, we checked if the first position after the stop codon with a PLP above the threshold matches a known polyadenylation site. More precisely, the prediction was considered a true positive (TP) if its position occurred at most 40 bases upstream of the 5' end of a documented site (shown in Figure S1). Predictions falling outside were considered false positives (FP) and sequences having no prediction exceeding the threshold were counted as false negatives (FN). As our specificity and sensitivity assessment relies on the assumption that all existing mRNA cleavage sites have been documented, we applied this procedure only to human 3'UTRs. For Drosophila, the coverage of 3P tags is unlikely to be complete.

**Figure 6:**
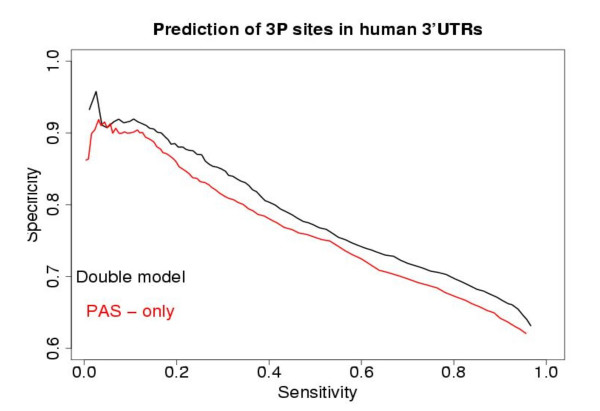
Sensitivity and specificity of mRNA CS prediction human 3'000 bases long 3'-UTRs for varying PLP thresholds using the double model shown in Figure 4A (black curve) compared to the PAS model (4B), (red curve). Sensitivity = TP/(TP + FN), Specificity = TP/(TP + TN).

The sensitivity and specificity analysis shows that incorporation of the DSE improves the prediction quality for all PLP thresholds tested (Figure 6). The increase in specificity due to the DSE is between 2 and 5%, and is relatively uniform as a function of sensitivity although it slightly increases at higher sensitivity (Figure 6A). For human 3'UTRs of 3000 bases, we achieved a specificity of 77% when setting the sensitivity to 50% (and 64% for a sensitivity of 90%). Legendre and Gautheret [9] expressed their results by comparing the predictions on 1000 bp long 3'UTRs to a series of control sets, such as shuffled 3'UTRs and intron sequences. For a sensitivity of 55%, they reported a specificity of 72 to 84% for their program depending on the control set, and 68 to 82% for the program of Tabaska and Zhang (1999). To avoid variation due to selection of the control set, we quantified our predictions by comparing them to experimentally documented polyadenylation sites. Using this strategy, we achieved specificity and sensitivity values comparable to those reported by Legendre and Gautheret, even though we scanned sequences that were three times longer.

## Discussion

### Sequence characteristics around the mRNA cleavage site

To investigate polyadenylation signals we used an exhaustive and unbiased database of CSs based on systematic mapping of all expressed sequences (cf. Methods) to genomes. Applying the same protocol to human and fly allowed pointing out strong similarities in the organization of their mRNA cleavage site regions. Based on these datasets, we identified PAS and DSE in both species, and confirmed the specificity and relevance of these signals for mRNA cleavage site prediction.

We defined as trustable CSs all genome-aligned EST reads containing a polyA sequence not present on the genome. Thus, our data differed from those assembled by [9] or [19] in two main aspects. First, we did not require the CS ESTs to overlap with a RefSeq, which allows us to take into account distant polyadenylation sites that might not be covered by mRNA sequences in RefSeq, which are very often incomplete at their 3' ends. Similarly, in [23], the candidate EST tags were derived from Unigene clusters, which might also counterselect distant downstream polyadenylation sites. Secondly CS were not selected for the presence of well-described PAS variants. In fact, scanning our CSs for the 13 common PAS variants [6] confirmed most of them and revealed very similar distributions in both species. The relative frequency of PAS motifs might reflect the efficacy of their recognition by the CSPF, and suggests that the specificity of this enzyme is well conserved between the two species. Interestingly, 15% of human mRNA cleavage sites (22% in *Drosophila*) do not have any of the previously described PAS motifs. Absence of over-represented signals in these suggests that cleavage might happen via an alternative mechanism. Compared to the numbers reported by Beaudoing at al [6], and Tian et al [23], we observe a lower frequency of all known polyadenylation variants, and a higher proportion of sequences without any known variant. We performed additional tests that ruled out a widespread contamination of our dataset with false priming sequences. Rather, detailed analysis suggests that the proportion of the main PAS variants increases with the number of tags documenting a PAS, so that the lower numbers that we observe are due to the more complete coverage of our dataset. Consistently with what was found in [25], the fine structure of the features observed in the nucleotide profiles in human mRNA CS region is largely conserved in *Drosophila*, while studies of CSs in *C. elegans *[15] and *S. cerevisiae *(reviewed in [3]) showed different profiles. Recent phylogenetic studies confirm that arthropods are closer to chordates than to nematodes [20]. Our observation is thus consistent with this finding, and may indicate that CS organization in human and *Drosophila melanogaster *sequences evolved before separation of arthropods and chordates.

The similarities between human and *Drosophila *mRNA CS regions led us to search for finer sequence motifs. We consistently identified the well-known PAS motif AAUAAA and a weaker U-rich motif that we interpreted as the DSE. Our consensus sequence for the human DSE (Figure 3C) is U [U/G]UCU [G/U]U. Tabaska and Zhang identified a similar DSE using a much smaller dataset and a motif-search tool (gibbs-seq) searching for two copies per fragment, while we required only one occurrence per sequence. A recent detailed study focusing on motifs involved in alternative polyadenylation [24] reports 4 conserved UG-rich motifs in the DSE regions, one of them (CDE.1) is very similar to the motif we found in all polyadenylation site tags. DSE weight matrices derived from human 3UTRs with one of the four main PAS variants [see Additional file 1, table 1] are extremely similar, although they slightly more polarized in the sequences lacking the AAUAAA variant. Our refined DSE motif, a U-rich motif with conserved G residues, confirms the presence of a DSE in Drosophila, as suggested earlier in word-searches using a smaller set of ESTs [25]. Its similarity with the human DSE is consistent with observations that crucial residues for RNA binding in C-terminus of the RNA-binding domain of CstF-64 are conservatively substituted in *Drosophila *[10]. It is possible that the lower information content of the DSE as compared to the PAS reflects true biological variability of DSE, important for the control of alternative polyadenylation. Variability in DSE motifs might influence this process as it was postulated that the presence of sub-optimal DSE sequences causes a differential choice of polyadenylation sites depending on the expression level of CstF protein [10]. We investigated the relationship between the PAS and DSE motifs, but we do not see significant difference between the DSE WMs derived from sequences with different PAS variants [see Additional file 1, table 2]. However, the DSE motifs could be tissue-specific. Different consensus sequences might be bound by tissue-specific isoforms of CstF-RNA binding subunits [21] or competitor proteins [22].

### Prediction of 3' mRNA termination

We used the derived PAS and DSE motifs to build a probabilistic model of human and *Drosophila *mRNA CSs. Previously constructed tools for human CS prediction used a quadratic discriminant function [8] or were based on RNA secondary structure [9]. Both tools require the presence of the main AAUAAA or AUUAAA variants of the polyadenylation motif. We chose the HMM framework, which makes our approach suitable for easy incorporation in gene prediction programs[13]. Similar approaches have been used for prediction of mRNA cleavage in yeast [14] and *C. elegans*[15], with more complex models of the polyadenylation region including 4 to 6 distinct subsequent signal regions interspersed with higher order background models. Our model consists of the PAS and DSE weight matrices chained together by a Gaussian zero-th order background spacer reflecting the distance between the 2 motifs (Figure 4A). We have verified that higher order background models did not change the predictions significantly. In both human and *Drosophila*, inclusion of DSE elements in HMMs increased the accuracy of the model predictions. However, our model does not include a signal for the CS itself. These putative signals cover only one or two bases, and have variable locations with respect to both PAS and DSE (microvariability in the CS [12]). Using CS signals did not improve our predictions. We assessed the performance of the model in human sequences using documented mRNA cleavage sites and our model performed better in mRNA CS recognition than previously reported tools (cf. Figure 5). In test sequences containing 3'000 bp downstream of the translation stop codon, we achieved a specificity of 70% for a sensitivity of 80%. Notice that we did not require the presence of the main variants of the PAS. However, as this matrix is more informative than the DSE, PLP scores are dominated by the PAS and sites strongly differing from the consensus will tend to be missed (false negatives, [see Additional file 1, Figure 1]). So even though our prediction specificity is high, differentially weighting the PAS and DSE might lead to further improvements. In fact, the reported specificity might be underestimated, since our evaluation procedure assumed that all existing polyadenylation sites are documented with 3' tags. This probably results in our overestimation of false positives. One extension to improve our current model would be to exploit comparative genomics to define more precise DSE weight matrices, which should be possible for both mammals and insects.

## Conclusion

We analyzed the sequence region encompassing mRNA cleavage site in human and Drosophila ESTs, derived PAS and DSE motifs for both species and proved their specificity. We integrated these motifs into a HMM which predicts mRNA cleavage and polyadenylation sites with higher specificity than previously published tools.

Moreover, we show that the sequence regions involved in polyadenylation are highly conserved between these two species. Our study underlines the value of using the primary sequence data derived from EST projects, as well as genome sequences, for the large-scale documentation and analysis of polyadenylation sites. With the constantly increasing number of available ESTs, future studies might uncover sequence signals that control tissue specific regulation of alternative polyadenylation.

## Methods

### Polyadenylation tags for human and Drosophila melanogaster

We mapped all publicly available human (described in [16]; data available at our ftp site [26]) and *Drosophila *EST reads (246'248 EST sequences from BGDP EST collection, raw sequencing data, obtained from Dr. Mark Stapleton, BGDP) to the corresponding genomes. The sequences that matched the genome with at most 2 mismatches and ended with at least 10 A's not present in the genome were taken to be derived from *bona fide *polyadenylation events. The genome sequences spanning from -50 to + 50 bp relative to the CS ("3' tags") were collected for all polyadenylation events. Micro-variability of a few bases in the position of the CS is frequently observed [Additional file 1, Figure 2A] consistent with [12]. All unique CS sites so defined were used for the statistics in Fig. 1, 2, 3 and for building the PAS and DSE models. A polyadenylation site can be defined as a cluster (our 3P clusters) of closely spaced CS, for which the cleavage is driven by the same PAS. Therefore CS that differ only by a few nt (cf. Fig S2A) are clustered for the evaluation of polyadenylation sites (Fig 6) as in ref [12].

Figure 1A shows the distribution of the number of 3' tags per gene. In both human and *Drosophila*, a large fraction of genes are represented by a small number of tags, while some human genes have up to 5000 tags. Due to large quantity of data (590'008 tags), the distribution of human 3' tags is likely to reflect biological variation in the expression levels of the corresponding genes. For *Drosophila*, we were able to collect only 11'385 3' tags, because most of the sequences produced by the BGDP project were 5' ESTs. Although the average coverage is about 1/20^th ^of that in human, the similarity in these distributions in both species suggests that the *Drosophila *tags reflect similar expression variability. The clustered 3' tags documented a total of 53'469 polyadenylation regions in human, and 2'659 in *Drosophila*. All datasets used in the various sections are described and available at [27].

### Motif search and HMM algorithms

We searched for motifs in the 100-bp polyadenylation regions documented by clusters of 3' tags using the *meme *software [17]. To incorporate all sequences for defining the motifs, we applied preliminary *meme *runs to identify the two strongest signals (AAUAAA for PAS, UUUGUUU for human DSE, and UUUCUGU for fly DSE) which were then used as seeds to exhaustively search all sequences in groups of 250.

In fly the DSE seed is related although not identical with the hexamers founds in [25]. Hits in these runs were then used to define the final matrices if they met positional criteria. For the PAS hits we retained all occurrences with first nucleotide at positions between 20 and 38 (Figure 3). For the second motif, we retained all occurrences between nucleotide 51 and 73. The extension of the matrices was defined by the information content (IC=2+∑LpLlog⁡2(pL)
 MathType@MTEF@5@5@+=feaafiart1ev1aaatCvAUfKttLearuWrP9MDH5MBPbIqV92AaeXatLxBI9gBaebbnrfifHhDYfgasaacH8akY=wiFfYdH8Gipec8Eeeu0xXdbba9frFj0=OqFfea0dXdd9vqai=hGuQ8kuc9pgc9s8qqaq=dirpe0xb9q8qiLsFr0=vr0=vr0dc8meaabaqaciaacaGaaeqabaqabeGadaaakeaacqWGjbqscqWGdbWqcqGH9aqpcqaIYaGmcqGHRaWkdaaeqbqaaiabdchaWnaaBaaaleaacqWGmbataeqaaOGagiiBaWMaei4Ba8Maei4zaC2aaSbaaSqaaiabikdaYaqabaGccqGGOaakcqWGWbaCdaWgaaWcbaGaemitaWeabeaakiabcMcaPaWcbaGaemitaWeabeqdcqGHris5aaaa@416C@) at each position. Positions with IC > 0.2 bits, which provided a clear separation from surrounding background, were retained.

HMMs were trained and decoded using the implementation written by A. Krogh (unpublished). Training is based on the Baum-Welch expectation minimization procedure and models were decoded using either the Forward or posterior label probability (PLP) [18] algorithms. The PLP of the first nucleotide in the PAS motif at each position in the sequence is computed for polyadenylation site prediction. The full model architecture is shown in figure 4.

### HMM parameter optimization

When assessing the specificity of the DSE matrices, the only parameters that were optimized were the background emission probabilities (b1 = b2 = b3 for model 4A and 4A and b1 = b3 for 4B) and transitions (p1 and p3 in both models), while the weight matrices and Gaussian spacer parameters were kept fixed. After optimization, the model was decoded (Forward) on two sets of sequences: one set of positives independent from the training set, and a shuffled version thereof serving as negative controls. A model consisting of a single zero order background state was optimized on the training sequence and used as background score.

For prediction, the model was optimized using an iterative procedure rather than simultaneous optimization of all parameters using Baum-Welch. First we noticed that optimizing the background transitions p1 and p3 tended to place the cleavage site too far downstream, leading to inferior performance. Thus, p1 and p3 were scanned and fixed at values of optimal prediction. In the final model, these corresponded to an average length in the first background state of 500 in human. Spacer length [40] and SD [11] were always fixed.

### 3'UTR sequences used for model assessment

Since our purpose was to predict 3' mRNA end formation knowing the position of the stop codon, we restricted our set to genes with unique stop codons having at least one entry in RefSeq and one polyadenylation site. To avoid ambuiguites in the evaluation of predictions, we further restricted the sets to sequences having an unique mRNA cleavage site, documented spread on less 40 bp. These sets consisted of 7743 genes in humans and 1680 in *Drosophila*. The sets were randomly split in disjoint halves for model training and testing.

## Authors' contributions

CI, PB and CVJ created the 3P tags databases, FN designed the study, DR, FN and PB performed the sequence analysis, DR and FN created and optimized the 3P UTR prediction program, DR, FN and CVJ wrote the manuscript. All authors read and approved the final manuscript.

## Supplementary Material

Additional File 1 Statistics on variants of the polyadenylation signal and associated downstream element variants; Exemple of HMM prediction output. Click here for file

## Figures and Tables

**Table 1:** Frequency (%) of PAS motifs reported by Beaudoing *et al*. in the 50 bp upstream of documented mRNA cleavage sites for 7000 human genes (2048 fly genes).

**PAS motif**	**Human**	***Drosophila***	**PAS motif**	**Human**	***Drosophila***
AAUAAA	46.73	47.76	AAUACA	1.75	2.86
AUUAAA	16.19	10.3	GAUAAA	1.64	0.62
UAUAAA	4.32	4.53	AAGAAA	1.02	0.31
AGUAAA	4.00	2.50	AAAAAG	0.96	0.21
UUUAAA	2.51	1.20	AAAACA	0.61	0.31
AAUAUA	2.38	5.05	AAUAGA	0.60	0.57
CAUAAA	2.32	1.30	none	14.96	22.48

The variants with most different frequencies are underlined.

**Table 2:** Nucleotide frequency of PAS and DSE profiles.

		*Human*				*Drosophila*			
	*pos*	*%A*	*%C*	*%G*	*%U*	*%A*	*%C*	*%G*	*%U*

***PAS***	*1*	92.1	2.26	1.36	4.26	96.86	1.43	0.06	1.63
	*2*	74.72	0.54	4.57	20.14	85.96	0.58	1.76	11.68
	*3*	1.76	1.02	3.25	93.94	0.19	3.52	0.13	96.14
	*4*	98.43	0.15	1.36	0.04	98.04	0.52	1.3	0.13
	*5*	96.67	2.49	0.28	0.55	88.31	5.09	0.13	6.46
	*6*	99.46	0.18	0.18	0.17	99.47	0.19	0.19	0.13

***DSE***	*1*	8.72	6.62	10.52	74.13	2.04	12.26	8.07	77.6
	*2*	1.72	18.64	37.31	42.3	3.57	7.77	34.25	54.39
	*3*	4.94	20.65	9.25	65.15	4.49	13.19	29.65	52.65
	*4*	1.52	68.43	14.13	15.89	10.32	18.91	40.79	29.95
	*5*	8.66	0.15	0.00	91.16	5.41	10.53	21.47	62.57
	*6*	0.11	7.63	59.4	32.85	6.13	4.6	25.97	63.29
		9.08	20.42	22.58	47.9	1.22	26.17	19.52	53.06
