# Supplementary material for: Similarities and differences of polyadenylation signals in human and fly
Source: BMC Genomics. 2006 Jul 12;7:176. doi: 10.1186/1471-2164-7-176 (PMC1574307; doi:10.1186/1471-2164-7-176)
Supplement: Additional File 1 — Statistics on variants of the polyadenylation signal and associated downstream element variants; Exemple of HMM prediction output. [file 1471-2164-7-176-S1.doc]

Supplementary figures and tables

**Supplementary Table 1:** Nucleotides frequencies in human DSE derived from 3’ UTRs with different PAS variants. The DSEs following less common variants of the PAS are slightly more polarized, but altogether extremely similar to each other.

| **PAS variant** | | **Human DSE** | | | |
| --- | --- | --- | --- | --- | --- |
|  |  | **A** | **C** | **G** | **U** |
| **AAUAAA** | 1 | 9.15% | 7.90% | 14.00% | **68.96%** |
|  | 2 | 3.18% | 19.43% | 38.18% | 39.21% |
|  | 3 | 5.75% | 21.91% | 9.63% | 62.71% |
|  | 4 | 1.38% | **68.36%** | 12.81% | 17.45% |
|  | 5 | 6.98% | 0.45% | 0.00% | **92.57%** |
|  | 6 | 0.15% | 5.72% | **63.74%** | 30.39% |
|  | 7 | 8.47% | 22.76% | 26.20% | 42.58% |
|  |  |  |  |  |  |
| **AUUAAA** | 1 | 10.95% | 8.44% | 11.71% | **68.90%** |
|  | 2 | 3.27% | 19.17% | 35.73% | 41.83% |
|  | 3 | 5.12% | 20.42% | 10.13% | 64.32% |
|  | 4 | 1.36% | **64.54%** | 14.32% | 19.77% |
|  | 5 | 8.06% | 0.60% | 0.00% | **91.34%** |
|  | 6 | 0.16% | 5.94% | **63.18%** | 30.72% |
|  | 7 | 8.22% | 22.82% | 24.62% | 44.34% |
|  |  |  |  |  |  |
| **AGUAAA** | 1 | 10.29% | 8.24% | 14.41% | **67.06%** |
|  | 2 | 1.76% | 17.06% | 40.29% | 40.88% |
|  | 3 | 5.88% | 24.41% | 9.12% | 60.59% |
|  | 4 | 0.88% | **71.47%** | 9.41% | 18.24% |
|  | 5 | 8.53% | 0.00% | 0.00% | **91.47%** |
|  | 6 | 0.00% | 6.47% | **62.94%** | 30.59% |
|  | 7 | 7.06% | 28.24% | 21.18% | 43.53% |
|  |  |  |  |  |  |
| **UAUAAA** | 1 | 7.00% | 7.29% | 12.54% | **73.18%** |
|  | 2 | 4.37% | 17.20% | 37.03% | 41.40% |
|  | 3 | 4.96% | 17.49% | 6.12% | 71.43% |
|  | 4 | 2.33% | **67.35%** | 12.83% | 17.49% |
|  | 5 | 8.16% | 0.00% | 0.00% | **91.84%** |
|  | 6 | 0.00% | 4.66% | **63.56%** | 31.78% |
|  | 7 | 7.29% | 22.74% | 19.24% | 50.73% |
|  |  |  |  |  |  |
| **Other** | 1 | 7.39% | 7.65% | 14.35% | **70.62%** |
|  | 2 | 2.87% | 19.61% | 35.44% | 42.08% |
|  | 3 | 4.52% | 23.11% | 9.46% | 62.91% |
|  | 4 | 0.96% | **71.47%** | 11.53% | 16.05% |
|  | 5 | 8.13% | 0.48% | 0.00% | **91.39%** |
|  | 6 | 0.11% | 7.49% | **64.82%** | 27.58% |
|  | 7 | 7.70% | 21.36% | 24.39% | 46.55% |

AAUAAA : 6237 sequences

AUUAAA : 1949 sequences

AGUAAA : 402 sequences

UAUAAA: 419 sequences

Other motifs : 2950 sequences

**
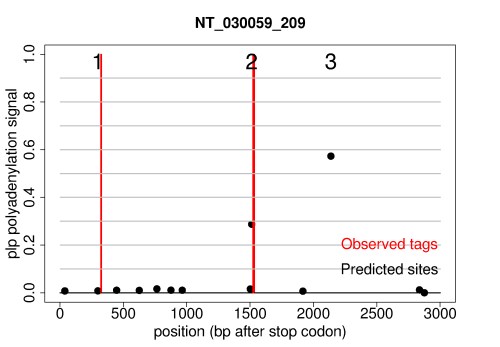
**

**Supplementary Figure 1**: Example of PLP values used for CS prediction. Black dots: predicted probability of polyadenylation signal. Red lines: position of observed 3’ tags. Our CS prediction for this gene is a false negative if the threshold is higher than 0.6, a false positive for a threshold between 0.3 and 0.6, and a true positive for a threshold < 0.33. PAS signal for the 1st 3’ tag = UAUAAA, PAS signal for the 2nd 3’ tag = AAUAAA.

**A.**

**B.
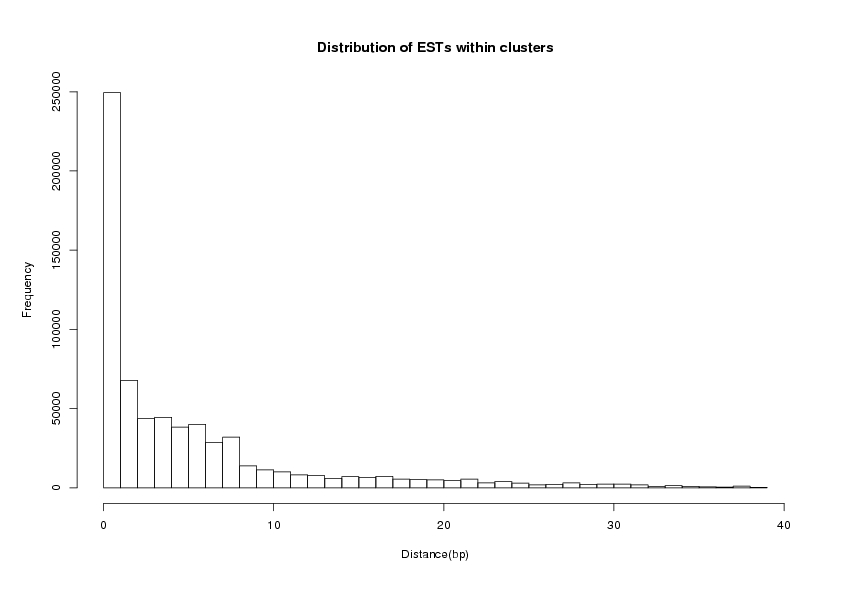
**


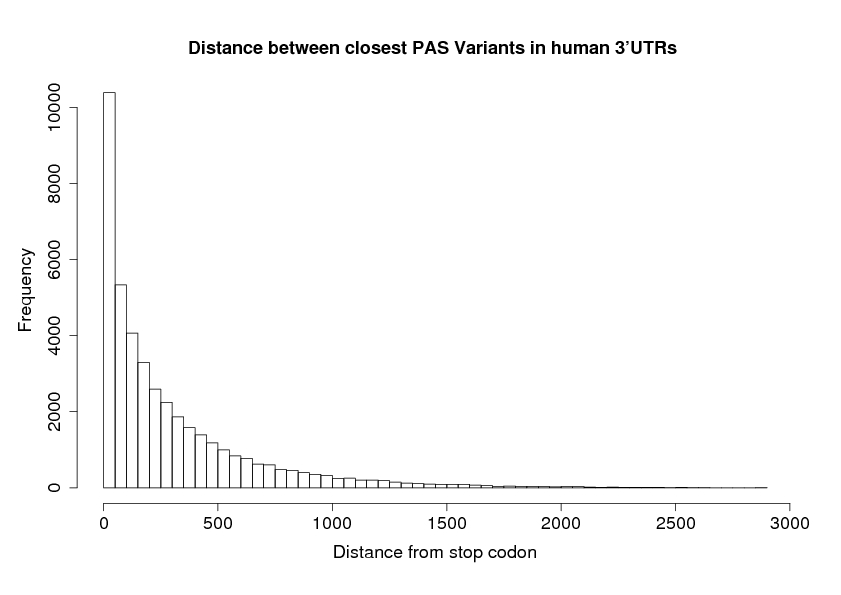


**Supplementary Figure 2:** A Relative position of ESTs within a human 3P cluster. B: Distance between the closest occurrences of the 4 major PAS variants (AAUAAA, AUUAAA, AGUAAA and UAUAAA) in human 3’UTRs. 3512 of 42094 distances (8.34%) are closer than 10 bp in human sequences, and 278 / 2535 (10.97%) in Drosophila sequences.
